# Effectiveness of self-management interventions in reducing cancer treatment-related cardiotoxicity in breast cancer survivors: A systematic review

**DOI:** 10.1016/j.apjon.2026.100931

**Published:** 2026-03-02

**Authors:** Mubei Yang, Qiaohong Yang, Gary Tse, Yuhua Ma, Qi-yuan Lyu, Wei Wang, Agnes Y. Lai, Yiheng Zhang, Carmen Wai Man Wong, Xianliang Liu

**Affiliations:** a School of Nursing and Health Sciences, Hong Kong Metropolitan University, Hong Kong, SAR, China; b School of Nursing, Jinan University, Guangzhou, China; c Department of Breast Surgery, The First Affiliated Hospital of Jinan University, Jinan University, Guangzhou, China

**Keywords:** Cancer therapy, Cardiotoxicity, Self-management intervention, Breast cancer survivors, Systematic review

## Abstract

**Objective:**

To evaluate the effectiveness of self-management interventions in reducing cancer therapy-induced cardiotoxicity among breast cancer survivors.

**Methods:**

Six English-language databases were searched for studies from January 2004 to November 2024. JBI appraisal tools and the Grading of Recommendations, Assessment, Development and Evaluation (GRADE) framework were applied to assess the methodological quality and certainty of the evidence. Meta-analysis and descriptive qualitative synthesis were applied.

**Results:**

A total of 11 randomised controlled trials (RCTs) involving 950 participants were included. A total of 516 participants (54%) were allocated to self-management intervention (e.g., aerobic and/or resistance exercise) groups, while 434 participants (46%) were assigned to control/usual care groups. The most frequently assessed cardiotoxicity-related parameters were cardiorespiratory fitness, cardiac function, and biomarkers, all of which may be attenuated by exercise. The meta-analysis indicated that exercise interventions significantly improved VO_2peak_ (MD = 2.71, 95% CI 1.23 to 4.20, *P* < 0.001) and left ventricular ejection fraction (MD = 1.80, 95% CI 0.06 to 3.54, *P* = 0.043), although heterogeneity was substantial. However, the effectiveness of exercise on secondary outcomes remains uncertain, the GRADE framework rated the certainty of the evidence as very low.

**Conclusions:**

Exercise has emerged as the most frequently employed self-management strategy for mitigating therapy-induced cardiotoxicity among breast cancer survivors. Evidence has indicated that structured exercise may help attenuate cancer therapy-induced cardiotoxicity, with VO_2peak_ emerging as a more sensitive marker than left ventricular ejection fraction. Nonetheless, further rigorously designed RCTs are warranted to solidify the evidence base and evaluate exercise interventions’ long-term sustainability and broader health benefits.

**Systematic review registration:**

PROSPERO: CRD42024621854.

## Introduction

Cancer remains a major global health challenge and a leading cause of mortality. An estimated 2.3 million new cases and 670,000 deaths from female breast cancer were reported in 2022.^1^ While the primary aim of cancer therapy is to eradicate disease or delay recurrence to prolong survival, breast cancer is increasingly managed as a chronic condition. Consequently, greater attention is warranted to the long-term sequelae of treatment, particularly cardiotoxicity. Both traditional and new targeted treatments—including chemotherapy, endocrine therapy, and radiation—can cause both early and delayed cardiotoxicity^2^^,^^3^ in part because they intersect with key cardiovascular signalling pathways.^4^ Reported incidence varies widely (approximately 6% to 39%), reflecting differences in agents, dose and duration, radiation fields, and patient susceptibility.^5^, ^6^, ^7^, ^8^, ^9^

The European Society of Cardiology defines cardiotoxicity as including health conditions such as cardiac dysfunction, myocarditis, vascular toxicity, arterial hypertension, and cardiac arrhythmias.^10^ Cardiac dysfunction is classified into asymptomatic and symptomatic types, with asymptomatic dysfunction assessed through left ventricular ejection fraction (LVEF), myocardial global longitudinal strain (GLS), and cardiac biomarkers, while symptomatic dysfunction, indicative of heart failure (HF), manifests with symptoms such as ankle swelling, breathlessness, and fatigue.^10^ Pre-existing cardiovascular risk substantially modifies the likelihood and severity of treatment-related cardiotoxicity.^11^ Compared with individuals without a cancer history, survivors have higher risks of cardiovascular mortality and of developing cardiovascular disease (CVD) risk factors, including hypertension and diabetes.^12^, ^13^, ^14^ These risks are compounded by shared lifestyle factors—poor diet, physical inactivity, obesity, and tobacco use.^15^

Cardiotoxicity and CVD are closely interconnected: pre-existing CVD can increase susceptibility to treatment-induced cardiotoxicity, while cardiotoxicity may accelerate the onset or progression of CVD. Once present, cardiotoxicity can interrupt or limit cancer therapy through HF or arrhythmias, diminish oncologic efficacy, and lead to long-term cardiovascular complications that impair quality of life (QoL) and survival.^16^ Recent advances in diagnostic techniques, including cardiac imaging and biomarker analysis, have enhanced the ability to detect subclinical cardiac dysfunction, suggesting the true burden may be underestimated.^17^ Given this dual morbidity and the potential for progressive decline, effective self-management strategies are essential to support cardiovascular health across survivorship.^18^

The concept of “self-management” originates from the Cognitive Learning Theory and positions individuals as active agents in their care.^19^ Self-management interventions typically include goal setting; health education; symptom monitoring; establishing routines that promote healthy behaviours; and iterative adjustment based on feedback and health status.^20^^,^^21^ Such programmes shift emphasis from illness to wellness, building skills in problem-solving, decision-making, resource use, and collaboration with clinicians.^22^ In the context of treatment-related cardiotoxicity, self-management encompasses non-pharmacological strategies such as physical activity, structured exercise, dietary modification, and optimisation of daily activity.^23^ For example, a randomised controlled trial (RCT) found that an exercise-based cardio-oncology rehabilitation programme helped to prevent a decline in LVEF during breast cancer (BC) chemotherapy.^24^ Yet only 30%–52% of cancer survivors adhere to physical activity recommendations,^25^ highlighting the need for structured support to embed these behaviours in daily life. Self-management interventions can provide that support, addressing adherence barriers and helping to bridge the gap between survivor needs and the capacity of health services.^26^

Previous systematic reviews have examined the effects of self-management interventions on physical and psychological health outcomes in cancer survivors.^27^, ^28^, ^29^ However, these reviews indicated that it was difficult to draw definitive conclusions regarding the impact of different types of self-management programmes on breast cancer survivors.^27^, ^28^, ^29^ Additionally, none of those reviews addressed treatment-related cardiotoxicities or cardiovascular health outcomes in evaluating self-management interventions. To address this gap, the present systematic review aimed to evaluate the effectiveness of self-management interventions specifically targeting cardiotoxicity and cardiovascular health issues arising from cancer therapy in breast cancer survivors.

## Methods

This systematic review was conducted according to the JBI methodology for systematic reviews of effectiveness^30^^,^^31^ and the 2020 PRISMA Statement for reporting systematic reviews and meta-analyses. The systematic review protocol has been registered in PROSPERO (CRD42024621854). This systematic review was conducted in accordance with the registered protocol, with no amendments or deviations made.

### Conceptual and operational definition

#### Self-management interventions

Self-management interventions are structured, non-pharmacological strategies that equip individuals to actively monitor, prevent, and manage their health conditions.^19^ Core components typically include goal setting; health education; symptom monitoring; establishment of routines that support healthy behaviours; and iterative adjustment based on feedback and health status.^20^^,^^21^ In this systematic review, eligible interventions were non-pharmacological programmes (e.g., educational, behavioural, or lifestyle-based) undertaken by cancer survivors to prevent or manage treatment-related cardiotoxicity or to improve cardiovascular health.

#### Breast cancer survivors

Individuals diagnosed with BC are considered survivors from the time of diagnosis and throughout the remainder of life.^32^

### Eligibility criteria

The inclusion and exclusion criteria were structured using the PICO framework—Participants, Intervention, Comparator, and Outcome—to ensure alignment between the review question, search strategy, study selection, and data extraction.^33^

#### Participants

Eligible participants were adults (aged ≥ 18 years) diagnosed with BC, regardless of gender or cancer therapy received.

#### Intervention

Self-management interventions aimed at preventing or managing cancer therapy–related cardiotoxicity, such as lifestyle-based interventions (e.g., supervised and non-supervised regular exercise), psychological interventions (e.g., maintaining a diary for mental health), social support (e.g., joining support groups), and educational programmes (e.g., education from health care professionals).^22^

#### Comparators

The eligible comparators included standard care, usual care, no intervention, or alternative approaches.

#### Outcomes

The primary outcome was cancer treatment-related cardiotoxicity, defined as myocardial injury or dysfunction identified by abnormalities in cardiac imaging (e.g., left ventricular ejection fraction [LVEF], global longitudinal strain [GLS]), deviations in cardiac biomarkers, or clinical symptoms of cardiovascular conditions.^10^^,^^16^ Abnormalities in cardiac imaging includes (1) a reduction in LVEF of at least 10% to an absolute value below 53%, and/or (2) a decrease of over 15% in GLS from baseline.^5^^,^^10^ The eligible secondary outcomes included broader cardiovascular health indicators (e.g., blood pressure [BP], lipid profiles, arrhythmia incidence), fatigue, psychological factors (e.g., anxiety, depression), and overall QoL.

Only RCTs were eligible for inclusion. Studies published in English were included. Studies published as conference abstracts, protocols, or without peer review were excluded.

### Search strategy

Two reviewers (MB Y and XL L) independently conducted comprehensive research using tailored MeSH terms and keywords in English-language databases and trial registers, including CINAHL, PubMed, Web of Science, Cochrane CENTRAL, PsycINFO, and EMBASE. The search strategy was guided by the PICO framework. The research included the following keywords for each database: “cancer”, “cancer survivor”, “self-care”, “self-management”, “symptom”, “experience”, “supportive care”, “cardiovascular abnormalities”, and “cardiotoxicity”. To reflect the emergence of cardio-oncology and structured self-management approaches, searches were limited to January 2004 through November 2024. Despite earlier work on cardiac rehabilitation in heart failure populations (e.g., from 2006),^34^ studies specifically evaluating self-management for cancer therapy–related cardiotoxicity remain sparse. Furthermore, the reviewers collaborated with a university librarian to create a comprehensive search strategy. For the search strategy example, see Appendix 1. Clinical trial registries (e.g., ClinicalTrials.gov) were searched to identify grey literature. Reference lists from all included studies, as well as those from similar systematic reviews, were carefully examined to identify additional eligible studies.

### Study selection

Study screening was conducted using Covidence. All citations and abstracts identified through the search strategy were systematically uploaded to Covidence. Duplicates, as well as any papers deemed irrelevant, were carefully identified and removed from the collection. Two reviewers (MB Y and XL L) independently examined the titles and abstracts based on predefined inclusion criteria. If further appraisal was required, the full texts were retrieved. During this process, any disagreement between the two researchers was resolved through discussion.

### Data extraction

Two reviewers (MB Y and XL L) independently extracted the data for each eligible study using Covidence. Extracted data items included author (year), region, study design, cancer stage, sample size and age, intervention and control group characteristics, outcomes related to cancer treatment-induced cardiotoxicity and cardiovascular health, and data collection timing. Additional variables extracted included intervention type, procedure, delivery personnel, presence of self-practice (yes/no), timing, duration, frequency, and follow-up period.

### Quality appraisal of the included studies

Two reviewers (MB Y and XL L) independently conducted a methodological quality appraisal of the included studies using a standardised critical appraisal instrument for RCTs from the JBI.^31^ The appraisal criteria encompassed study designs, methodologies, outcome measures, bias, and limitations. Any disagreements between the reviewers were resolved through discussion, with a third party involved if necessary. All studies deemed eligible for inclusion underwent data extraction and synthesis, irrespective of their assessed methodological quality. Furthermore, the methodological quality of all included studies factored into the interpretation of the results.

### Data synthesis

Data synthesis was conducted according to the guidelines of the JBI methodology for systematic reviews of effectiveness.^30^^,^^31^ Meta-analyses for pooled effectiveness were carried out using statistical software R 4.3.3. Statistical heterogeneity was assessed using the *I*-squared (*I*^*2*^) statistic test: a) 0%–40% indicates minimal heterogeneity, b) 30%–60% indicates moderate heterogeneity, c) 50%–90% indicates substantial heterogeneity, and d) 75%–100% indicates considerable heterogeneity. When the *I*^*2*^ value exceeded 50%, we utilized the random-effects model's results. Subgroup analyses were planned and conducted based on the interventions. When the data reported from the included studies could not be pooled, the results were synthesized used descriptive analysis.

Descriptive analysis was used to summarise the characteristics of the included studies, such as countries or regions where they were conducted, cancer types, participant completion rates, most frequently observed parameters, and timing of outcome assessments. Additionally, descriptive analysis detailed the self-management protocols, highlighting key aspects such as intervention type, procedure, instructor, timing, duration, frequency, and follow-up. Extracted data were summarised using a narrative subgroup approach. For each outcome domain (e.g., abnormalities in cardiac imaging, deviations in cardiac biomarkers, or clinical symptoms of cardiovascular conditions), results were described within each outcome domain and then synthesised into summary conclusions by the reviewers.

### Assessing certainty in the findings

The Grading of Recommendations, Assessment, Development and Evaluation (GRADE) approach was used to grade the certainty of the evidence, and a Summary of Findings was created using GRADEPro GDT software (McMaster University, ON, Canada).^35^ The Summary of Findings provided comprehensive information, including absolute risk values, estimates of relative risk, and a ranking of the evidence quality, which was based on considerations of bias risk, heterogeneity, and publication bias within the review results.

## Results

### Selection of studies

**Fig. 1:**
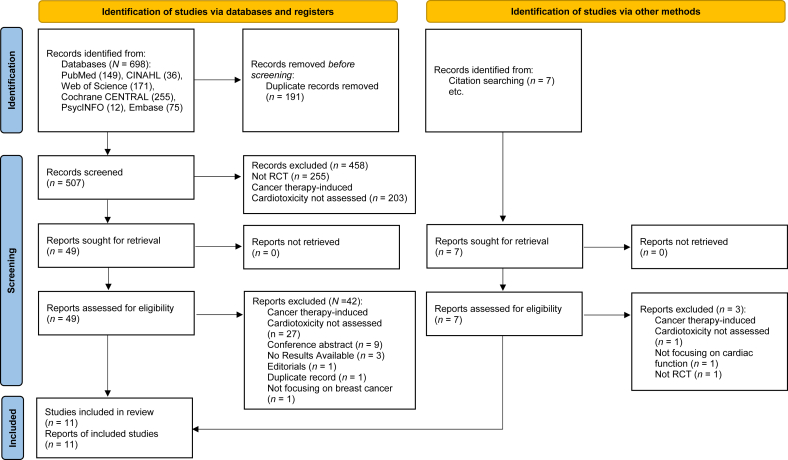
Flow diagram illustrating the original process of screening and identification of studies.

A total of 705 relevant records were identified through initial searches across six databases (*n* = 698) and other sources (manual retrieval, *n* = 7). After removing 191 duplicates and 458 records during title and/or abstract screening in Covidence, 56 records were deemed eligible for full-text review (Fig. 1). Of these, 11^24^^,^^36^, ^37^, ^38^, ^39^, ^40^, ^41^, ^42^, ^43^, ^44^, ^45^ met the inclusion criteria and were included in this systematic review.

### Study characteristics

Table 1 presents the following characteristics of the included studies. The 11 included studies were published between 2018 and 2024,^24^^,^^36^, ^37^, ^38^, ^39^, ^40^, ^41^, ^42^, ^43^, ^44^, ^45^ all in English. The included studies were conducted in various countries and regions: Australia (*n* = 1), Canada (*n* = 2), Portugal (*n* = 1), Spain (*n* = 1), Thailand (n = 1), France (*n* = 1), Sweden (*n* = 1), Poland (*n* = 1), India (*n* = 1), and Taiwan, China (*n* = 1). All 11^24^^,^^36^, ^37^, ^38^, ^39^, ^40^, ^41^, ^42^, ^43^, ^44^, ^45^ included RCTs recruited a total of 950 cancer survivors; all participants were BC patients at various stages. The completion rates for the intervention group (IG) and control group (CG) were 82.9% and 80.4%, respectively. The sample sizes across the RCTs ranged from 22 to 122 participants, with only two trials including more than 100 participants.^24^^,^^37^ The participants across the included studies were adult women ranging in age from 18 to 75 years, with reported mean and median ages consistently clustering within the middle-aged bracket of approximately 45–55 years.

**Table 1:** Characteristics of included studies.

Author (year)	Region	Study design	Cancer stage	Sample size and age [year, (Mean SD)]	Intervention group (IG)	Control group (CG)	Outcomes measures (related to this systematic review topic only)	Outcomes (related to this systematic review topic only)	Data collection timing
Foulkes et al. (2023)	Australia	RCT	Stage I to III BC	104 women, 40–75 years	Mobile app-guided aerobic and resistance exercise	UC: Standard oncology care with encouragement to maintain physical activity after therapy.	- CRF: VO_2peak_ - Cardiac function: LVEF, GLS, VO_2peak_ ≤ 18.0 mL/(kg·min) - Biomarkers: BNP, troponin - Cardiac reserve: left/right ventricular ejection fraction	- ExT improved VO_2peak_ by 3.5 mL/(kg·min） at 12 months - Enhanced cardiac output, stroke volume, and left/right ventricular ejection fraction reserve. No significant changes in LVEF or GLS. - No changes in BNP in either group. Post-chemotherapy troponin increased less in ExT than in UC.	At baseline (before or within 2 weeks of initiating AC), at 4 weeks after the completion of AC (4 months), and at 12 months after starting AC.
Antunes et al. (2023)	Portugal	RCT	Early-stage BC	93 women, mean age ∼50 years	Aerobic and resistance exercise	UC	- CRF: VO_2peak_ - Cardiac function: LVEF, GLS - Biomarkers: hs-TnT, NT-proBNP	- Ext increased VO_2peak_ by 3.1 mL mL/(kg·min） at 3 months post-AC. - No significant between-group differences in LVEF or GLS at the end of AC. - No significant effect on levels of NT-proBNP and hs-TnT.	Baseline, end of AC, and 3 months post-AC.
Díaz-Balboa et al. (2024)	Spain	RCT	Stage I–III BC	122 women, mean age 48.8 years	Aerobic and resistance exercise	UC: Physical activity advice provided via telephone every 2 months; monitoring for cardiotoxicity and cardiovascular disease risk factors every 3 months.	- CRF: VO_2peak_ - Cardiac function: LVEF, GLS - Biomarkers: NT-proBNP, troponin I	- VO_2peak_ remained unchanged in both groups. - LVEF decline attenuated in CORe group (mean difference −1.5%, *P* = 0.006). No changes in GLS - No changes in cardiac biomarkers.	Baseline and 2 weeks post-chemotherapy completion.
Siripanya et al. (2023)	Thailand	RCT	Stage I–II BC	30 women, 40–60 years	Aerobic exercise: Buddhist walking meditation	Non-exercising CG: UC without prescribed exercise, receiving only routine clinical therapy.	- CRF: VO_2peak_ - Cardiac function: stroke volume and cardiac output - Biomarkers: hs-CRP, MDA, IL-6 - Vascular reactivity: FMD, arterial stiffness (baPWV)	- FMD and VO_2peak_ decreased after AC in both groups; post-intervention, FMD and VO_2peak_ improved in the walking meditation group but remained lower in the CG. - Arterial stiffness increased in the CG but did not change in the IG. - No significant changes in MDA and IL-6. hs-CRP increased in both groups after AC, IG had lower hs-CRP levels than CG.	Baseline (pre-chemotherapy), pre-intervention (2 weeks post-chemotherapy initiation), and post-intervention (12 weeks).
Jacquinot et al. (2022)	France	Phase II RCT	HER2-positive BC	89 women, median age 51 years	Aerobic exercise	Standard care with trastuzumab treatment without a supervised exercise program.	- CRF: VO_2peak_, maximal power - Cardiac function: LVEF, GLS	- Peak VO_2_ increased by 2.6 mL/(kg·min） (95% CI, 1.8–3.4) in the IG, while no change was observed in the CG. - LVEF and GLS remained stable and showed no differences between groups.	Baseline (T0), 3 months (T3), 6 months (T6)
Ansund et al. (2021)	Sweden	RCT	Stage I–IIIa BC	240 women, 18–70 years	Aerobic and resistance exercise: RT-HIIT group and AT–HIIT group	UC: Written exercise recommendations per American College of Sports Medicine guidelines.	- CRF: VO_2peak_ - Biomarkers: NT-proBNP, cTnT	- VO_2peak_ was maintained in RT-HIIT and AT-HIIT but declined in UC; at 2 years, a decline in VO_2peak_ correlated with elevated biomarkers (cTnT > 10 ng/mL, NT-proBNP > 100 ng/mL). - cTnT increased in all groups post-intervention. NT-proBNP levels were lower in the IG at 1 year compared to CG.	Baseline, post-intervention (16 weeks), 1 year, 2 years and 5 years (5 year-assessments are currently ongoing and not included in this study) post-baseline.
Hojan et al. (2020)	Poland	RCT	HER2-positive BC	68 women, mean age 54.5 ± 6.05 years	Aerobic and resistance exercise	General physical activity recommendations, no supervised exercise.	- CRF: 6MWT - Cardiac function: LVEF, GLS - Biomarkers: MYO, hs-CRP, CK, CK-MB, IL-6	- 6MWT distance decreased significantly in CG but remained stable in IG. - LVEF decrease was mitigated in IG (no significant changes observed), whereas CG showed significant decreases (*P* < 0.05). No significant changes in GLS. - No significant changes in biomarkers.	Baseline (3–6 months post trastuzumab initiation) and post-intervention
Kirkham et al. (2023)	Canada	RCT	Stage I–III BC	77 women, mean age 52 ± 10 years	Aerobic and resistance exercise	UC: standard cancer care without a structured exercise or dietary program.	- CRF: VO_2peak_ - Cardiac function: LVEF, GLS - Biomarkers: Troponin I, BNP	- VO_2peak_ was higher. - LVEF unchanged at 52 weeks (61% ± 6%) in both groups. No group differences in GLS. - No group differences in biomarkers.	Baseline (prechemotherapy), end of chemotherapy (24 weeks), and post-intervention (52 weeks)
Inbaraj et al. (2023)	India	RCT	Stage I–III BC	68 participants, mean age 45.4 ± 7.7 years (TAU), 46.3 ± 6.1 years (TAUYT)	Aerobic exercise	UC	- Cardiac Function: RHR and HRV (RMSSD, SDNN, pNN50, LF, HF, LF/HF ratio)	- After chemotherapy, patients in the CG had higher RHR, lower HRV, increased sympathetic indices (LF power), and sympathovagal imbalance (LF/HF) compared to the IG.	Baseline (pre-chemotherapy), after 6 cycles of chemotherapy (post-intervention)
Kirkham et al. (2018)	Canada	RCT	Stage I–III BC	27 women, mean age 50 ± 9 years	Aerobic exercise	UC: No vigorous exercise within 72 hours prior to and 48 h after chemotherapy; allowed to perform light-to-moderate physical activity.	- Cardiac function: LV longitudinal strain, twist, LVEF, stroke volume and cardiac output - Biomarkers: NT-proBNP, cTnT - Hemodynamics: RHR, blood pressure	- No significant changes in longitudinal strain, twist, LVEF. - NT-proBNP and cTnT significantly increased, with no significant group difference.	Baseline (0–14 before AC), and 7–14 days after the final AC
Chung et al. (2020)	Taiwan, China	RCT	Stage I-III BC	32 women, mean age 50.3 years (control) / 52.4 years (exercise)	Aerobic and resistance exercise	UC	- CRF: VO_2peak_ - Cardiac function: LVEF	- The IG demonstrated higher exercise capacity (VO_2_ 12.1 mL/(kg·min) *vs* 13.6 mL/(kg·min), *P* < 0.05). - The CG showed lower cardiac systolic function (LVEF 62% *vs* 70%, *P* < 0.05).	Baseline, 3, 6, and 12 months post-chemotherapy

RCT, Randomized Controlled Trial; BC, breast cancer; UC, Usual care; IG, Intervention group; CG, Control group; AC, anthracycline chemotherapy; CRF, cardiorespiratory fitness; RHR, Resting heart rate; HRV, Heart rate variability; cTnT, Cardiac Troponin T; baPWV, brachial–ankle pulse wave velocity; FMD, flow-mediated dilation; hs-CRP, High-Sensitivity C-Reactive Protein; MDA, Malondialdehyde; IL-6, Interleukin-6; 6MWT, 6-Minute Walk Test; CK, creatine kinase; CK-MB, Creatine Kinase Myocardial Band; MYO, Myoglobin; hsTnT, high sensitivity Troponin T; NT-proBNP, NT-pro-brain natriuretic peptide; BNP, B-type natriuretic peptide; RT-HIIT, resistance and high intensity interval training; AT–HIIT, aerobic and high-intensity interval training; LV, Left Ventricular; LVEF, Left Ventricular Ejection Fraction; GLS, Global Longitudinal Strain; VO_2peak_, Peak Oxygen Consumption; ExT, exercise training; CORe, Cardio-Oncology Rehabilitation; TAU, Treatment as Usual; TAUYT, Treatment as Usual with Yoga Therapy; RMSSD, root mean square of differences between adjacent normal-to-normal intervals; pNN50, percentage of the number of pairs of adjacent normal-to-normal interval difference by > 50 ms; LF, low frequency; HF, high frequency.

The most frequently observed parameters related to cancer therapy-induced cardiotoxicity in these 11 studies^24^^,^^36^, ^37^, ^38^, ^39^, ^40^, ^41^, ^42^, ^43^, ^44^, ^45^ were impaired cardiac function (*n* = 10)^24^^,^^36^, ^37^, ^38^, ^39^, ^40^^,^^42^, ^43^, ^44^, ^45^ and CRF (*n* = 9),^24^^,^^36^, ^37^, ^38^, ^39^, ^40^, ^41^, ^42^, ^43^ followed by cardiac biomarkers (*n* = 8).^24^^,^^37^, ^38^, ^39^^,^^41^, ^42^, ^43^^,^^45^ The most commonly used instruments were LVEF and GLS for assessing cardiac function (*n* = 8),^24^^,^^36^, ^37^, ^38^^,^^40^^,^^42^^,^^43^^,^^45^ VO_2peak_ for assessing CRF (*n* = 8),^24^^,^^36^, ^37^, ^38^, ^39^, ^40^, ^41^^,^^43^ and BNP and/or troponin for assessing biomarkers (*n* = 6).^24^^,^^37^^,^^38^^,^^41^^,^^43^^,^^45^

Most outcomes were assessed both before and immediately after the intervention.^36^, ^37^, ^38^, ^39^, ^40^, ^41^^,^^43^^,^^44^ Six studies conducted follow-up assessments post-intervention, ranging from seven days to five years.^24^^,^^36^^,^^38^^,^^40^^,^^41^^,^^45^ Additionally, only one study conducted an extra assessment during the intervention.^37^

#### Intervention protocols

Table 2 systematically summarises the intervention protocols, outlining intervention types, procedures, instructor details, self-practice details, timing, durations, frequencies, and follow-up periods. Exercise was the most commonly used self-management intervention for reducing therapy-induced cardiotoxicity in cancer survivors. The interventions were categorised into two main types: aerobic exercise and resistance exercise. All 11 studies^24^^,^^36^, ^37^, ^38^, ^39^, ^40^, ^41^, ^42^, ^43^, ^44^, ^45^ implemented aerobic interventions, with seven studies incorporating both aerobic and resistance exercises.^24^^,^^36^, ^37^, ^38^^,^^41^, ^42^, ^43^ Additionally, five studies progressively increased exercise intensity throughout the intervention period.^37^, ^38^, ^39^^,^^42^^,^^43^

**Table 2:** Intervention protocol of included studies.

Author (year)	Type	Procedure	Instruction trainer	Self-practice (Y/N)	Timing	Duration	Frequency	Follow-up
Foulkes et al. (2023)	Aerobic and resistance exercise	- Phase 1: 12-week supervised exercise, 3 days/week during AC. - Phase 2: 14-week semisupervised exercise, 4 days/week after AC (2 supervised, 2 unsupervised). - Phase 3: 26-week unsupervised maintenance, 4 days/week with remote support and 6 face-to-face reviews.	Oncology team	Y	During and after AC	12 months	3–4 days/week	NA
Antunes et al. (2023)	Aerobic and resistance exercise	- Aerobic exercise: 1. Stage 1: During the first 2 weeks, patients did 20 min of aerobic training at < 50% HR reserve. 2. Stage 2: For every 2 weeks, the duration increased by 3 min until 30 min was reached, with patients exercising at 65%–80% HR reserve - Resistance exercise: 1. Stage 1: During the first 2 weeks, patients performed 2 sets of 10 repetitions per exercise with minimal or no resistance. 2. Stage 2: If no adverse events or symptoms occurred, resistance was added, allowing patients to perform 3 sets of 12 RM. 3. Stage 3: After completing 3 sets of > 12 RM for 3 consecutive sessions, a 5%–10% load increase was considered.	Exercise physiologist and physiotherapist	N	Druing AC: From 1 to 2 days after the first cycle of AC	20–24 weeks	3 sessions/week, Receiving AC every 2 weeks: 60 exercise sessions; every 3 weeks: 72 exercise sessions	3 months
Díaz-Balboa et al. (2024)	Aerobic and resistance exercise	- One-hour session - Monitoring for cardiotoxicity every 3 months using the cardio-onco-hematology unit - Strength training with body weight and elastic bands - Aerobic exercise at 50%–85% of HR reserve	Physiotherapist	N	During the chemotherapy	3–12 months	2 days/week	2 weeks
Siripanya et al. (2023)	Aerobic exercise: Buddhist walking meditation	− 30 min/session - Before the exercise program, detailed face-to-face instructions on walking meditation were provided - A 25 beats/min audio rhythm was played on an MP3 recorder, with the words “Budd” and “Dha” voiced while squeezing rubber balls rhythmically in participants' both hands - Phase 1 (weeks 1–6): walking exercise was performed at 41%–50% HRR for 30 min (3 intervals of 10 min with 3-min rest) 3 times per week - Phase 2 (weeks 7–12): walking meditation exercise was performed at 51%–60% HRR for 45 min (3 intervals of 15 min with 3-min rest) 3 times per week.	Not mentioned	Y	During AC	12 weeks	3 times/week	NA
Jacquinot et al. (2022)	Aerobic exercise	− 36 sessions, 55 min/session - Each session began with 5 min warm-up at 1⁄2 VT_1_, followed by 9 work bouts of 5 min each, totaling 45 min of exercise. - Each 5-min work bout consisted of 4 min at moderate intensity (“base”) followed by 1 min at high intensity (“peak”). - At the end of the last “peak,” a 5-min active recovery was performed at 1⁄2 VT_1_, the same power as the warm-up.	Adapted physical activity specialist	N	During adjuvant trastuzumab	12 weeks	3 sessions/week	3 months
Ansund et al. (2021)	Aerobic and resistance exercise	- RT-HIIT group: 1. Resistance training (8–12 reps at 75%–80% of 1RM) targeting major muscle groups. 2. Followed by 3 × 3 min bouts of aerobic HIIT on a cycle ergometer. - AT–HIIT group: 1. Each session with 20 min of moderate-intensity continuous aerobic exercise. 2. Followed by the same HIIT regimen as the RT-HIIT group.	Exercise physiologist or oncology nurse	N	During chemotherapy	16 weeks	2 times/week	1 year, 2 years, and 5 years
Hojan et al. (2020)	Aerobic and resistance exercise	- Started 3–6 months after trastuzumab treatment - Aerobic exercise: 1. Daily sessions: 45–50 min, including 2 min warm-up, 45 min of aerobic activities, and 3 min relaxation. 2. Activity options: Brisk walking, treadmill running, or cycling (up to two forms per session). - Resistance exercise: 1. Daily session (5 days/ week) lasting 40–45 min. 2. Isometric, concentric, and eccentric training with 1–3 sets of 8–10 reps targeting the trunk, upper body, and leg muscles in various positions. 3. Progressive intensity using elastic bands (1–4.5 kg), TRX system, indoor rower, dumbbells, and medicine balls, adjusted by movement amplitude, exercises, or concentric velocity.	Physiotherapist and therapist	N	After 3–6 months of trastuzumab	9 weeks	5 days/week	NA
Kirkham et al. (2023)	Aerobic and resistance exercise	− 60–90 minutes/session - Aerobic and resistance intensity progressed every 4 weeks, as tolerated. - Aerobic exercise: 1. Home-based exercise: Walking and cycling, 1–2 days/week if possible. 2. Supervised aerobic exercise: 10–60 minutes, based on participant goals and home-based exercise 3. Aerobic intensity: 60%–90% of maximal heart rate or RPE 3–8 (Borg scale 0–10). 4. Aerobic modes: Cycle ergometer, elliptical, or treadmill. - Resistance exercise: 1. Resistance training: 2 sets of 10–15 reps for 8 whole-body exercises and 2 core strength exercises. 2. Resistance intensity: RPE 3–8 (Borg 0–10 scale), based on prior experience.	Exercise physiologist	Y	During and after trastuzumab and/or AC	52 weeks	Up to 2 sessions/week	NA
Inbaraj et al. (2023)	Aerobic exercise	− 40-min sessions every morning - Contents: Yoga therapy, including asanas (postures), pranayama (breathing exercises), meditation, and relaxation techniques. - The initial 1-week yoga session: Delivered in person as a one-to-one session in the hospital setting. - After discharge following the first chemotherapy cycle, participants were instructed to continue practicing yoga at home using a video provided by the study team. - For subsequent chemotherapy cycles (every 21 days for 6 cycles), participants received in-person yoga sessions and practice adjustments at the hospital.	Yoga therapists	Y	During chemotherapy (6 cycles)	18 weeks	5 days/week	NA
Kirkham et al. (2018)	Aerobic exercise	− 4 sessions - Supervised treadmill exercise: 1 session, 24 h pre-AC (22–26 h before doxorubicin) - Session structure: 10-min warm-up, 30 min at 70% HRR, 5-min cool-down - Abstained from vigorous aerobic exercise 72 h pre- and 48 h post-treatment.	Not mentioned	N	24 h pre-AC (before each chemothrapy session).	4 bouts (1 per chemotherapy cycle)	1 session/ chemotherapy cycle	7–14 days
Chung et al. (2020)	Aerobic and resistance exercise	− 24 sessions - Each training session: 40 min aerobic, 15 min resistance, 5 min flexibility - Exercise started with chemotherapy and was paused during week 2 of each cycle until ANC reached 500 cells/mm^3^. - Aerobic exercise: 1. 5 min warm-up at 50% HRR, 30 min at 70%–80% HRR, 5 min cool-down at 50% HRR. 2. Performed on a stationary ergometer or treadmill. - Resistance exercise: 1. 10–20 reps per set at RPE 13–14, 2–3 sets, totaling 15 min. 2. Targeted upper and lower extremities (elbow flexion, shoulder abduction, squats, hip movements) with elastic band (intermediate tension). - Flexibility training: 1. Each exercise was held for 30 s per set, with 2 sets per session. 2. Included self-stretching of pectoralis muscles, soft tissue mobilization, shoulder mobilization, and static stretching of cuff muscles, quadriceps, and hamstrings.	Physical therapists	N	During chemotherapy	3 months	2–3 sessions/week	3 months, and 9 months

AC, anthracycline chemotherapy; Y, Yes; N, No; NA, Not Applicable; HR, heart rate; HRR, heart rate reserve; VT1, Ventilatory Threshold 1; RT-HIIT, resistance and highintensity interval training; AT–HIIT, aerobic and high-intensity interval training; RM, Repetition Maximum; RPE, Rating of Perceived Exertion; ET, Exercise training; CPET, Cardiopulmonary Exercise Testing; ANC, absolute neutrophil count.

All 11 studies^24^^,^^36^, ^37^, ^38^, ^39^, ^40^, ^41^, ^42^, ^43^, ^44^, ^45^ specified the types of aerobic exercise performed, including treadmill,^24^^,^^36^, ^37^, ^38^^,^^42^^,^^45^ elliptical trainer,^37^ stationary bike,^38^ stepping,^38^ Buddhist walking meditation,^39^ cycle ergometer,^24^^,^^40^^,^^41^ walking,^42^^,^^43^ cycling,^42^^,^^43^ yoga,^44^ and stationary ergometer.^36^

Most of the interventions were supervised or instructed by experts, except for two studies that did not specify the supervisors or instructors.^39^^,^^45^ The most commonly reported supervisors included physiotherapists,^24^^,^^38^^,^^42^ exercise physiologists,^38^^,^^41^^,^^43^ oncology team members or nurses,^37^^,^^41^ adapted physical activity specialist,^40^ therapist,^42^ yoga therapist,^44^ and physical therapist.^36^ Four studies included unsupervised self-practice exercise sessions.^37^^,^^39^^,^^43^^,^^44^

Regarding timing, eight studies were conducted during treatment,^24^^,^^36^, ^37^, ^38^, ^39^, ^40^, ^41^^,^^44^ one before treatment,^45^ one after treatment,^42^ and one both before and after treatment.^43^ The most common intervention durations were three months^24^^,^^36^^,^^39^^,^^40^ and 12 months.^24^^,^^37^^,^^43^ Most of the studies^24^^,^^36^, ^37^, ^38^, ^39^, ^40^, ^41^, ^42^, ^43^, ^44^ implemented interventions two to five days per week, except for one study that scheduled one session per chemotherapy cycle,^45^ with the most common frequencies being three sessions per week^36^, ^37^, ^38^, ^39^, ^40^ and two sessions per week.^24^^,^^36^^,^^41^

Three studies^24^^,^^37^^,^^38^ had to adapt their intervention protocols due to the COVID-19 pandemic: one study^37^ transitioned the exercise model from supervised to home-based; one study^38^ conducted most of the treadmill exercise stress tests without gas analysis due to limitations; and one study^24^ delivered exercise sessions via videoconference and estimated VO_2peak_ using the 6MWT.

### Methodological quality of included studies

The appraisal results are presented in Table 3. Three studies^37^^,^^38^^,^^43^ were rated as having a low risk of bias, seven studies^24^^,^^36^^,^^39^^,^^40^^,^^42^^,^^44^^,^^45^ had a moderate risk of bias, and one study^41^ had a high risk of bias. All 11 studies ^24^^,^^36^, ^37^, ^38^, ^39^, ^40^, ^41^, ^42^, ^43^, ^44^, ^45^ reported proper randomisation, including computer/software-generated randomisation and third-party randomisation,^45^ although three studies^24^^,^^41^^,^^44^ did not specify the exact randomisation methods.

**Table 3:** Methodology quality of the included studies.

Study	Internal Validity Bias										Statistical conclusion validity			Total of “Yes” scores (%)	Risk of bias
	Selection and allocation			Administration of intervention/exposure			Assessment, detection, and measurement of the outcome			Participant retention					
	Q1	Q2	Q3	Q4	Q5	Q6	Q7	Q8	Q9	Q10	Q11	Q12	Q13		
Foulkes et al. (2023)	Y	N	Y	N	N	Y	Y	Y	Y	Y	Y	Y	Y	11/13, 84.6%	Low
Antunes et al. (2023)	Y	Y	Y	N	N	Y	Y	Y	Y	Y	Y	Y	Y	11/13, 84.6%	Low
Díaz-Balboa et al. (2024)	Y	Unclear	Y	N	N	Y	Y	Y	Y	Y	Y	Y	Y	10/13, 76.9%	Moderate
Siripanya et al. (2023)	Y	Y	Y	N	N	Y	Y	Y	Y	Y	Unclear	Y	Y	10/13, 76.9%	Moderate
Jacquinot et al. (2022)	Y	Unclear	Y	N	N	Y	Y	Y	Y	Unclear	N	Y	Y	8/13, 61.5%	Moderate
Ansund et al. (2021)	Y	Unclear	Y	N	N	Y	Unclear	Y	Y	Unclear	N	Y	Y	7/13, 53.8%	High
Hojan et al. (2020)	Y	Y	Y	N	N	Y	Y	Y	Y	Unclear	N	Y	Y	9/13, 69.2%	Moderate
Kirkham et al. (2023)	Y	Y	Y	N	N	Y	Y	Y	Y	Y	Y	Y	Y	11/13, 84.6%	Low
Inbaraj et al. (2023)	Y	Y	Y	N	N	Y	Y	Y	Y	Unclear	N	Y	Y	9/13, 69.2%	Moderate
Kirkham et al. (2018)	Y	Y	Y	N	N	Y	Y	Y	Y	Unclear	N	Y	Y	9/13, 69.2%	Moderate
Chung et al. (2020)	Y	Y	Y	N	N	Y	Y	Y	Y	Unclear	Y	Y	Y	10/13, 76.9%	Moderate

Q1. “Was true randomization used for assignment of participants to treatment groups?” Q2. “Was allocation to treatment groups concealed?” Q3. “Were treatment groups similar at the baseline?” Q4. “Were participants blind to treatment assignment?” Q5. “Were those delivering treatment blind to treatment assignment?” Q6. “Were treatment groups treated identically other than the intervention of interest?” Q7. “Were outcomes assessors blind to treatment assignment?” Q8. “Were outcomes measured in the same way for treatment groups?” Q9. “Were outcomes measured in a reliable way?” Q10. “Was follow up complete and if not, were differences between groups in terms of their follow up adequately described and analyzed?” Q11. “Were participants analyzed in the groups to which they were randomized?” Q12. “Was appropriate statistical analysis used?” Q13. “Was the trial design appropriate, and any deviations from the standard RCT design (individual randomization, parallel groups) accounted for in the conduct and analysis of the trial?” ——Source: Barker, T.H., Stone, J.C., Sears, K., Klugar, M., Tufanaru, C., Leonardi-Bee, J., Aromataris, E. and Munn, Z., (2023). The revised JBI critical appraisal tool for the assessment of risk of bias for randomized controlled trials. JBI Evidence Synthesis, 21(3): 494–506.

Except for four studies,^24^^,^^37^^,^^40^^,^^41^ most of the studies reported the use of concealed allocation: five used opaque envelopes,^38^^,^^42^, ^43^, ^44^, ^45^ three employed a third party,^38^^,^^39^^,^^45^ and one used an online generator.^36^ Regarding baseline characteristics, all 11 studies^24^^,^^36^, ^37^, ^38^, ^39^, ^40^, ^41^, ^42^, ^43^, ^44^, ^45^ reported comparable characteristics between the groups.

Since all 11 studies^24^^,^^36^, ^37^, ^38^, ^39^, ^40^, ^41^, ^42^, ^43^, ^44^, ^45^ involved exercise as the intervention, neither the participants nor the intervention instructors were blinded to treatment assignment. Only one study did not clearly report whether the outcomes assessors were blinded.^41^ Five studies^24^^,^^36^, ^37^, ^38^^,^^43^ used intention-to-treat analysis.

The outcomes detailed in the Summary of Findings (Table 4) focused on the impact of self-management interventions on cardiotoxicity and cardiovascular outcomes when compared with control groups.

**Table 4:** Summary of findings.

Self-management interventions versus control for managing cancer therapy-induced cardiotoxicity					
Outcome	No. of Participants (Studies)	Follow-up Duration	Impact	Certainty of Evidence (GRADE)	Comments
**Cardiorespiratory Fitness (CRF)***(Ma*inly m*easured by VO*_*2peak*_*)*	9 studies, 855 participants	2 weeks to 5 years	Mixed results. Significant improvement in VO_2peak_ across multiple studies, except for one.	Moderate	Moderate confidence in the effect of exercise on CRF. While results were mixed, most studies showed consistent improvements in VO_2peak_, with some variations.
**Cardiac Function***(Mainly measured by LVEF and GLS)*	10 studies, 710 participants	1 week to 9 months	Mixed and inconsistent results. While exercise attenuated LVEF decline in some studies, no significant between-group differences were observed in GLS.	Moderate	Moderate confidence in the effect of exercise on cardiac function. Exercise attenuated LVEF decline in some studies, but results for LVEF and GLS were inconsistent.
**Biomarkers of Cardiotoxicity***(Measured by multiple biomarkers)*	8 studies, 761 participants	1 week to 5 years	No consistent effect on biomarkers. Many studies reported no changes, no significant changes, no differences, or no significant between-group differences.	Low	Low confidence in the effect of exercise on biomarkers of cardiotoxicity due to inconsistent findings and high variability in biomarker selection and assessment.
**Cardiovascular Health Indicators** Effect on BP, lipid profiles, and reduce arrhythmias	7 studies, 496 participants	1 week to 2 weeks	Mixed results. Most studies found no significant between-group differences in BP, except for one. Only one study assessed lipid profiles, reporting significant reductions in total cholesterol and LDL in the intervention group. No studies reported the incidence of arrhythmias.	Very low	Very low confidence in the effect of exercise on cardiovascular health indicators. Limited data exist, with only one study reporting LDL reductions and no studies assessing arrhythmias.
**Patient-Reported Outcomes** Effect on fatigue	3 studies, 191 participants	3 months	Only one study reported significant fatigue improvement after exercise.	Very low	Very low confidence in the effect of exercise on fatigue, as only one study provided supporting data and only one study conducted follow-up.
**Patient-Reported Outcomes** Effect on psychological well-being (anxiety, depression)	2 studies, 149 participants	1–2 weeks	One study found no significant effect on anxiety or depression, while another reported improved depressed mood but no change in anxiety.	Very low	Very low confidence in the effect of exercise on anxiety and depression due to small sample sizes and mixed findings across two studies.
**Patient-Reported Outcomes** Effect on QoL	2 studies, 119 participants	3 months	Only one reported significant QoL improvement.	Very low	Very low confidence in the effect of exercise on QoL, as only one study reported a significant improvement and only one study conducted follow-up.

CRF, Cardiorespiratory Fitness; VO_2peak_, Peak Oxygen Consumption; LVEF, Left Ventricular Ejection Fraction; GLS, Global Longitudinal Strain; QoL, Quality of Life; BP, Blood Pressure; LDL, Low-Density Lipoprotein.

GRADE Working Group grades of evidence

**High certainty:** We are very confident that the true effect lies close to that of the estimate of the effect.

**Moderate certainty:** We are moderately confident in the effect estimate: the true effect is likely to be close to the estimate of the effect, but there is a possibility that it is substantially different.

**Low certainty:** Our confidence in the effect estimate is limited: the true effect may be substantially different from the estimate of the effect.

**Very low certainty:** We have very little confidence in the effect estimate: the true effect is likely to be substantially different from the estimate of effect.

### Effects of exercise interventions

#### Primary outcome

The effects of the exercise interventions on the symptoms of BC survivors are summarised in Table 1, categorised into CRF, cardiac function, and biomarkers.

##### Effects on CRF

**Fig. 2:**
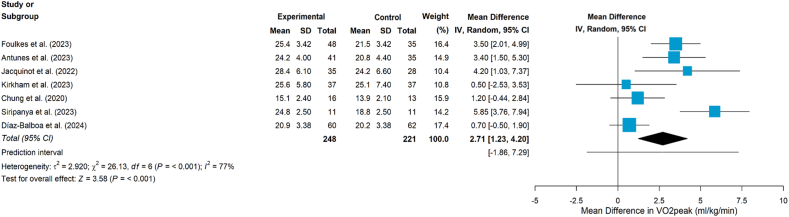
Effects on VO_2peak__. VO_2peak_, Peak Oxygen Consumption._

**Fig. 3:**
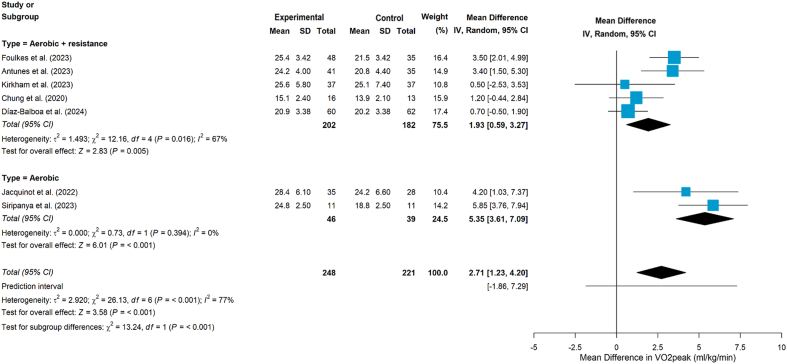
Subgroup analyses by intervention type (VO_2peak_). VO_2peak_, Peak Oxygen Consumption.

Nine studies^24^^,^^36^, ^37^, ^38^, ^39^, ^40^, ^41^, ^42^, ^43^ assessed the impact of aerobic and/or resistance exercise on CRF. Of the nine studies,^24^^,^^36^, ^37^, ^38^, ^39^, ^40^, ^41^, ^42^, ^43^ all but one study,^42^ which used the 6MWT, measured CRF using VO_2peak_.^24^^,^^36^, ^37^, ^38^, ^39^, ^40^, ^41^^,^^43^ Eight studies found that exercise improved VO_2peak_ compared with UC,^36^, ^37^, ^38^, ^39^, ^40^, ^41^, ^42^, ^43^ and one study reported no change in VO_2peak_ in either group.^24^ The meta-analysis indicated that exercise interventions significantly improved VO_2peak_ (MD = 2.71, 95% CI 1.23 to 4.20, *P* < 0.001, Fig. 2), although heterogeneity was substantia (*I*^*2*^ = 77%, *P* < 0.001). Subgroup analyses by intervention type (combined aerobic plus resistance training vs aerobic training alone; Fig. 3) showed statistically significant improvements in VO_2peak_ in both subgroups. Notably, the aerobic-only subgroup showed no evidence of heterogeneity (*I*^*2*^ = 0%, *P* = 0.394).

##### Effects on cardiac function

**Fig. 4:**
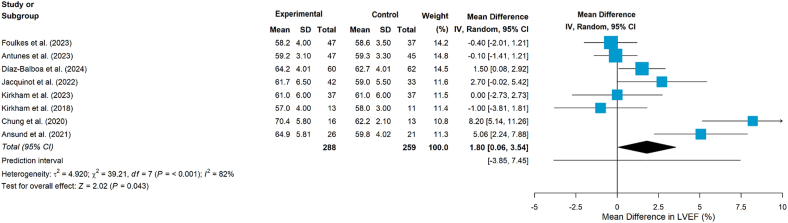
Effects on LVEF. LVEF, left ventricular ejection fraction.

**Fig. 5:**
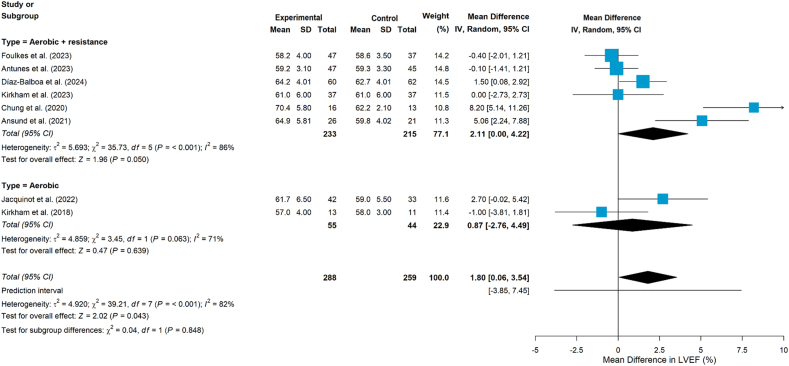
Subgroup analyses by intervention type (LVEF). LVEF, left ventricular ejection fraction.

**Fig. 6:**
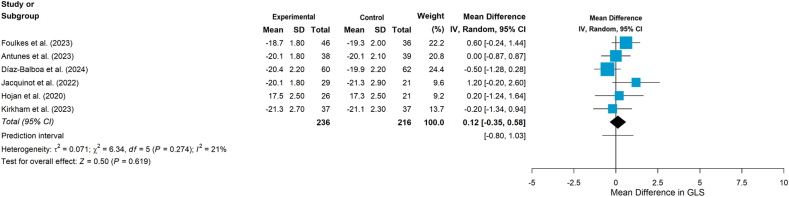
Effects on GLS. GLS, global longitudinal strain.

Ten studies^24^^,^^36^, ^37^, ^38^, ^39^, ^40^^,^^42^, ^43^, ^44^, ^45^ examined the impact of aerobic and/or resistance exercise on cardiac function. Of the 10 studies,^24^^,^^36^, ^37^, ^38^, ^39^, ^40^^,^^42^, ^43^, ^44^, ^45^ one study measured stroke volume and cardiac output,^39^ while another study assessed resting heart rate and heart rate variability (including RMSSD, SDNN, pNN50, LF, HF, and LF/HF ratio).^44^ The remaining studies used LVEF and/or GLS as measures.^24^^,^^36^, ^37^, ^38^^,^^40^^,^^42^^,^^43^^,^^45^ Moreover, one study^37^ additionally measured functional disability using VO_2peak_ ≤ 18.0 mL/(kg·min), which suggested that LVEF screening alone may be insufficient for detecting left ventricular systolic dysfunction. The pooled results showed that exercise interventions had a statistically significant effect on improving LVEF (MD = 1.80, 95% CI 0.06 to 3.54, *P* = 0.043, Fig. 4), although heterogeneity was substantial (*I*^*2*^ = 82%, *P* < 0.001). Subgroup analyses by intervention type (combined aerobic plus resistance training vs aerobic training alone; Fig. 5) did not demonstrate significant effects on LVEF in either subgroup (*P* = 0.050 and *P* = 0.639, respectively). In addition, the pooled results showed no statistically significant effects on GLS (MD = 0.12, 95 % CI −0.35 to 0.58, *P* = 0.619, Fig. 6).

##### Effects on biomarkers

Eight studies^24^^,^^37^, ^38^, ^39^^,^^41^, ^42^, ^43^^,^^45^ examined the impact of aerobic and/or resistance exercise on biomarkers. Six studies^24^^,^^37^^,^^38^^,^^41^^,^^43^^,^^45^ assessed BNP and/or troponin levels. One study^39^ measured hs-CRP, MDA, and IL-6, while another study^42^ analysed MYO, hs-CRP, CK, CK-MB, and IL-6.

Only two studies^37^^,^^39^ suggested that exercise mitigated the biomarker levels. One study^37^ found no changes in BNP, but post-chemotherapy troponin increased less in the IG. Similarly, one study^39^ reported no significant changes in MDA and IL-6, but hs-CRP was lower in the IG. Additionally, one study^41^ reported lower NT-proBNP levels in the IG at one year compared with the CG.

#### Secondary outcomes

##### Blood pressure

**Fig. 7:**
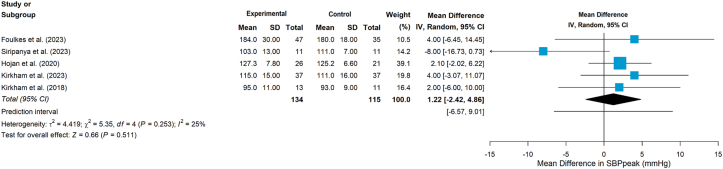
Effects on peak systolic blood pressure.

Seven studies assessed BP.^24^^,^^37^^,^^39^^,^^42^, ^43^, ^44^, ^45^ In three studies,^37^^,^^42^^,^^45^ the exercise intervention did not produce significant group differences. One study^24^ reported baseline BP only, while one study^44^ mentioned BP measurement but did not provide data. Two studies^43^^,^^46^ reported systolic BP reduction was greater in the CG, and one^43^ specifically reported that diastolic BP decreased similarly in both groups. Five included studies reported the peak systolic blood pressure, the meta-analysis results showed no statistically significant effects on peak systolic blood pressure (MD = 0.22, 95%CI −2.42 to 4.86, *P* = 0.511, Fig. 7).

##### Lipid profiles

Only one study^43^ assessed lipid profile changes, which found significant reductions in total cholesterol and LDL in the IG, with no significant changes in the CG.

##### Arrhythmias

No studies reported the incidence of arrhythmias.

##### Fatigue

Three studies^38^^,^^39^^,^^44^ examined exercise's effect on fatigue: one study^38^ found fatigue (38.2%) to be a common side effect of exercise training; one study^39^ reported significant fatigue improvement after exercise; and one study^44^ did not present fatigue data but noted it was a dropout reason. As these studies used different fatigue measures, a meta-analysis is not feasible.

##### Anxiety and depression

Two studies^24^^,^^45^ examined exercise's effect on anxiety and depression: one study^24^ found no significant differences in anxiety or depression between groups post-intervention; and the other study^45^ reported improvement in depressed mood but no significant differences in anxiety. As these studies used different anxiety and depression measures, a meta-analysis is not feasible.

##### QoL

Two studies^39^^,^^40^ assessed QoL changes: one study^39^ reported significant QoL improvement post-exercise, and the other study^40^ provided baseline QoL data but no post-intervention data.

##### Safety outcomes or adverse events

Among the included studies, three trials^41^, ^42^, ^43^ did not report safety outcomes. Six studies^24^^,^^36^^,^^39^^,^^40^^,^^44^^,^^45^ explicitly stated that no adverse events occurred during the exercise sessions; for example, one study^24^ found that the cardio-oncology rehabilitation programme was safe and well tolerated. Two studies^37^^,^^38^ reported minor exercise-related adverse events, including pre-existing musculoskeletal injuries,^37^ mild postural hypotension,^37^ generalised fatigue,^38^ pain in the extremities,^38^ and dizziness.^38^

## Discussion

### Main findings

This systematic review evaluated the effectiveness of self-management interventions for preventing or mitigating cancer therapy–induced cardiotoxicity in breast cancer survivors. Although exercise is established in cardiac rehabilitation,^47^^,^^48^ its specific effectiveness on cancer-related cardiotoxicity is less clear. Synthesizing 11 RCTs, we examined impacts on CRF, cardiac function, and biomarkers, and explored patient-centered outcomes including fatigue, mental health, and QoL.

Most trials reported clinically meaningful increases in VO_2__peak_ following aerobic or combined aerobic–resistance training, and the meta-analysis indicated that exercise interventions significantly improved VO_2peak_ (*P* < 0.001), consistent with prior reviews in oncology populations.^48^^,^^49^ However, heterogeneity in assessment, timing, and exercise intensity limited comparability. Several included studies^36^, ^37^, ^38^^,^^40^ demonstrated early improvements—some within four months—and short-term persistence post-intervention. These consistent gains suggest VO_2_peak is not only responsive to exercise but may serve as a more sensitive and clinically relevant marker of early cardiotoxic effects compared to imaging parameters.

Importantly, VO_2_peak reflects integrated cardiovascular and peripheral function, helping explain its superior sensitivity.^50^ In contrast, echocardiographic parameters such as LVEF and GLS are often insensitive to subtle myocardial dysfunction, especially in the context of heart failure with preserved ejection fraction (HFpEF), where ejection fraction may remain “normal” despite substantial diastolic and microvascular abnormalities.^51^^,^^52^ Thus, functional capacity—as indexed by VO_2__peak_—may offer greater prognostic utility and earlier detection of cardiotoxicity risk than traditional imaging, aligning with its recognition by the American Heart Association as a core clinical measure in heart failure.^53^

Findings on cardiac function were mixed. Among eight studies^24^^,^^36^, ^37^, ^38^^,^^40^^,^^42^^,^^43^^,^^45^ assessing LVEF or GLS, six^37^^,^^38^^,^^40^^,^^42^^,^^43^^,^^45^ reported no significant between-group differences. Subgroup analyses by intervention type (combined aerobic plus resistance training vs aerobic training alone) did not demonstrate significant effects on LVEF in either subgroup. This lack of effect may be attributable to measurement insensitivity, small sample sizes, or short intervention durations. Only two studies^37^^,^^39^ reported significant biomarker improvements (troponin, NT-proBNP), further highlighting the limited and inconsistent response of molecular markers in this context. One possibility is that these biomarkers reflect structural or inflammatory injury^54^ rather than dynamic functional capacity, and thus may not change with moderate-dose exercise interventions^55^ unless cardiotoxicity is already overt. This mismatch between functional and biochemical responsiveness highlights the need for integrated, multidimensional assessment frameworks in future trials.

Evidence for secondary outcomes (blood pressure, lipid profiles, arrhythmias, fatigue, anxiety, depression, and QoL) was sparse, inconsistent, and often underreported. Some trials deferred these results to separate publications or measured them using different tools. Moreover, most included participants were early-stage BC survivors with relatively preserved function, which may have limited the measurable impact on QoL. Another explanation is that while exercise improves physiological performance, psychological and symptomatic outcomes may require longer durations, multimodal support, or targeted psychological interventions to observe significant effects. Given the established benefits of exercise in reducing cancer-related fatigue and improving mental health and QoL in BC survivors,^56^^,^^57^ this represents a missed opportunity and signals the need for standardized, longitudinal assessment of patient-centered outcomes.

Our findings align with growing evidence that exercise may act as a cardioprotective intervention by preserving functional reserve rather than reversing structural injury.^58^ Several trials in our review adopted high-intensity interval or personalized aerobic programs—approaches known to enhance these mechanisms—yet effects on imaging and biomarkers remained limited, suggesting that functional adaptations may precede or even prevent detectable structural decline. This supports the rationale for prioritizing functional endpoints like VO_2_peak in both clinical care and trial design, especially in HFpEF-predominant populations such as female breast cancer survivors.^51^, ^52^, ^53^

The included studies predominantly enrolled middle-aged women (mean 45–55 years) with preserved functional status, generally excluding those with significant comorbidities. While this likely contributed to the favorable safety profile, it limits the generalizability of findings to older, frailer survivors or those with pre-existing cardiovascular disease. Future research must therefore prioritize tailored, age-appropriate interventions to ensure safety and efficacy across the broader survivorship spectrum.

### Methodological considerations and certainty of evidence

Most included studies demonstrated moderate to low risk of bias, with only one^41^ classified as high risk. The overall methodological quality was strengthened by consistent reporting of proper randomisation, allocation concealment, and appropriate statistical analyses, suggesting strong internal validity across the majority of trials. The GRADE assessment rated the evidence as moderate for CRF and cardiac function, and low for biomarkers. Lack of blinding, small sample sizes, and varied intervention durations contributed to the evidence's limitations. Still, the observed improvements in CRF and some cardiac measures provide moderate support for including exercise in self-management plans for cardiotoxicity prevention and rehabilitation.

### Limitations

This systematic review has some limitations. Only English-language studies were included, introducing potential language and publication bias and limiting generalizability to non–English-speaking contexts. This systematic review aimed to evaluate the full spectrum of self-management interventions designed to mitigate cancer treatment–related cardiotoxicity in breast cancer survivors. Although the search strategy was designed to capture diverse modalities—including dietary and psychological approaches—the comprehensive literature search yielded only studies utilizing exercise-based interventions. This finding identifies a significant gap in the literature and highlights the need for a broader array of self-management strategies to help breast cancer survivors manage treatment-related cardiotoxicity.

Moreover, we synthesised evidence from both supervised and unsupervised exercise programmes because exercise is fundamentally self-managed, even when health care professionals or trainers provide instruction and support goal setting, participants retain primary responsibility for undertaking and maintaining the prescribed activity. Accordingly, including both delivery formats was necessary to represent the breadth of exercise as a self-management strategy. Nevertheless, future research should prioritise the design and evaluation of more sustainable and empowering models—such as remote monitoring and feedback systems enabled by digital health technologies (e.g., mobile applications and wearable devices)—to better support long-term adherence and real-world implementation. Additionally, the very small number of included studies and their sparse distribution across cardiotoxicity outcomes (e.g., cardiac dysfunction and cardiorespiratory fitness), precluded the planned quantitative synthesis (meta-analysis) and necessitated reliance on narrative synthesis for most outcomes.

## Conclusions

Exercise has emerged as the most frequently employed self-management strategy for mitigating cancer therapy-induced cardiotoxicity among breast cancer survivors. Across 11 included trials, evidence suggests moderate-certainty improvements in CRF, while effects on cardiac function (e.g., LVEF, GLS) were mixed, likely reflecting variability in assessment methods and limited sensitivity of conventional imaging. VO_2_peak appears to be a more responsive indicator of early cardiotoxicity and a useful complement to echocardiographic measures. However, these findings should be interpreted with caution given the limited number of included studies and the very low to moderate certainty of the evidence as rated by the GRADE framework. Nurses can lead early cardiovascular risk screening and, in collaboration with exercise professionals, implement individualized, risk-stratified exercise prescriptions alongside patient education, self-monitoring, and symptom surveillance. Integrated within survivorship pathways, these practices can safely promote participation and may attenuate long-term cardiotoxicity, reinforcing the coordinating role of nursing in cardio-oncology care. Future trials should extend follow-up to evaluate durability, employ standardized outcomes and timing (including VO_2__peak_ and patient-reported measures), and examine broader or multimodal self-management strategies (e.g., dietary and psychological interventions, digital support). Attention to adherence, safety, equity, and cost-effectiveness will enhance generalizability and guide implementation.

## CRediT authorship contribution statement

MBY, QHY, YHM, AYL, GT, WW, QYL, YHZ, WMW, and XLL contributed to the study design. XLL and MBY contributed to drafting and revising the manuscript, as well as preparing all figures and tables for submission. QHY, YHM, AYL, GT, WW, QYL, YHZ, and WMW reviewed the manuscript and contributed guidance on methodological aspects. All authors read and approved the final manuscript.

## Ethics statement

Not required.

## Data availability

The authors confirm that the data supporting the findings of this study are available within the article.

## Declaration of generative AI and AI-assisted technologies in the writing process

No AI tools/services were used during the preparation of this work.

## Funding

This work is supported by the Hong Kong Metropolitan University Faculty Research Startup Fund (Grant No. FRSF/2024/01). The funders had no role in considering the study design or in the collection, analysis, interpretation of data, writing of the report, or decision to submit the article for publication.

## Declaration of competing interest

The authors declare no conflict of interest.
