# Supplementary material for: Effectiveness of self-management interventions in reducing cancer treatment-related cardiotoxicity in breast cancer survivors: A systematic review
Source: Asia Pac J Oncol Nurs. 2026 Mar 2;13:100931. doi: 10.1016/j.apjon.2026.100931 (PMC13054610; doi:10.1016/j.apjon.2026.100931)
Supplement: Multimedia component 1 [file mmc1.docx]

**Appendix 1: Search strategy**

**PubMed**

| # | Query | Results retrieved |
| --- | --- | --- |
| S1 | (((((((((((self-management[MeSH Terms]) OR (self care[MeSH Terms])) OR (Exercise[MeSH Terms])) OR (self-management[Title/Abstract])) OR (self care[Title/Abstract])) OR (supportive care[Title/Abstract])) OR (social support[Title/Abstract])) OR (health education[Title/Abstract])) OR (health promotion[Title/Abstract])) OR (exercise*[Title/Abstract])) OR (lifestyl*[Title/Abstract])) OR (psychosocial support*[Title/Abstract]) | 900320 |
| S2 | (((((((((((((cardiotoxicity[MeSH Terms]) OR (cardiovascular abnormality[MeSH Terms])) OR (cardiovascular disease[MeSH Terms])) OR (cardiotoxicit*[Title/Abstract])) OR (therapy-induced cardiotoxicit*[Title/Abstract])) OR (chemotherapy-induced cardiotoxicit*[Title/Abstract])) OR (cardiovascular toxicit*[Title/Abstract])) OR (cardiovascular abnormality[Title/Abstract])) OR (cardiovascular abnormaliti*[Title/Abstract])) OR (cardiovascular health[Title/Abstract])) OR (cardiovascular diseas*[Title/Abstract])) OR (heart health[Title/Abstract])) OR (cardiovascular effect*[Title/Abstract])) OR (cardiovascular function[Title/Abstract]) | 2961866 |
| S3 | (((cancer survivors[MeSH Terms]) OR (Neoplasms[MeSH Terms])) OR (cancer survivor*[Title/Abstract])) OR (oncology patients[Title/Abstract]) | 4045173 |
| S4 | S1 AND S2 AND S3 | 3965 |
| S5 | Filters applied: English, from 2004/1/1 to 2024/12/1, Randomized Controlled Trial | 149 |
